# Novel cellular immunotherapies for hematological malignancies: recent updates from the 2021 ASH annual meeting

**DOI:** 10.1186/s40164-022-00316-8

**Published:** 2022-09-24

**Authors:** Ji-nuo Wang, Tianning Gu, Yongxian Hu, He Huang

**Affiliations:** 1 Bone Marrow Transplantation Center, The First Affiliated Hospital, Zhejiang University School of Medicine, 79 Qingchun Road, Hangzhou, 310003 Zhejiang China; 2 Liangzhu Laboratory, Zhejiang University Medical Center, Hangzhou, China; 3 Institute of Hematology, Zhejiang University, Hangzhou, China; 4 Zhejiang Province Engineering Laboratory for Stem Cell and Immunity Therapy, Hangzhou, China

**Keywords:** Hematological malignancies, Cellular immunotherapy, The 2021 ASH annual meeting, Chimeric antigen receptor (CAR), Induced pluripotent stem cell (iPSC)

## Abstract

Cellular immunotherapy, including the chimeric antigen receptor T (CAR-T) cell therapy and CAR- natural killer (CAR-NK) cell therapy, has undergone extensive clinical investigation and development in recent years. CAR-T cell therapy is now emerging as a powerful cancer therapy with enormous potential, demonstrating impressive anti-tumor activity in the treatment of hematological malignancies. At the 2021 ASH annual meeting, numerous breakthroughs were reported concerning acute lymphocytic leukemia (ALL), lymphoma, acute myeloid leukemia (AML), and multiple myeloma (MM). Universal CAR-T cell and CAR-NK cell therapy, as well as induced pluripotent stem cell (iPSC)-derived immunotherapy, offer great “off-the-shelf” benefits. Major development and updates of cellular immunotherapy for hematological malignancies reported at the 2021 ASH annual meeting are summarized in this review.

## Background

Chimeric antigen receptor T (CAR‐T) cell therapy is a novel immunotherapy that directs genetically engineered T cells with specific target to antigen-expressing tumor cells [1]. With years of preclinical and clinical development, CAR-T cell therapy is now emerging as a desirable treatment strategy with a tolerable toxicity profile for many hematological malignancies [2]. So far, FDA have approved four CD19-targeted CAR-T cell products for refractory B cell malignancies, and two B cell maturation antigen (BCMA)-targeted CAR-T cell products for multiple myeloma (MM), which are provided for patients who relapse at least after four prior lines of treatment [3, 4]. However, we are still confronted with challenges including the lack of more suitable tumor-specific targets, short duration of CAR-T cells or CAR-natural killer (CAR-NK) cells, high production cost, and high relapse rate. Excitingly, researchers are making advances in new target design, functional enhancement, precise regulation, invention of universal CAR-T cell, and transformation of different chassis cells. This review summarized the latest updates of novel cellular immunotherapies for hematological malignancies from the 2021 ASH annual meeting.

### Updates of B-cell acute lymphoblastic leukemia (B-ALL) therapy from the 2021 ASH annual meeting

#### Real-world data for CD19-targeted CAR-T cell therapy for treatment of relapsed or refractory (R/R) B-cell acute lymphoblastic leukemia

Initially approved for R/R B-ALL in pediatric and young adult patients in April 2017 in the USA [2, 3], Tisagenlecleucel (Tisa-cel) is an autologous CD19-directed T-cell immunotherapy that has been widely used in many countries. Early real-world data for Tisa-cel from the Center for International Blood and Marrow Transplant Research (CIMBTR) registry reported high efficacy with tolerable adverse events (AEs) [5]. A non-interventional prospective study from the CIBMTR registry enrolled 451 patients aged ≤ 25y with R/R B-ALL who received commercial Tisa-cel since August 30, 2017 [6]. After a median follow-up of 21.5 months, the best overall response (BOR) of complete remission (CR) was 87.3% (95% confidence interval [CI], 83.1–90.7), of which 98.7% (148/150) were at negative minimal residual disease (MRD) status. Any-grade cytokine releasing syndrome (CRS) was observed in 58.0% (232/400) of patients, with grade ≥ 3 in 17.8% (71/400). Immune effector cell-associated neurotoxicity syndrome (ICANS) was observed in 27.3% (109/400) of patients, with grade ≥ 3 in 10.0% (40/400). Unfortunately, 20.5% (82/400) of patients died due to recurrence or progression of primary disease. Although early real-world evidence found lower cell dose and viability of CAR-T cells in commercial Tisa-cel product than those in previous pivotal trials, there was no apparent association with manufacturing parameters and clinical outcomes [5]. In addition, current prospective study identified greater disease burden and heavier pretreatment in young adult patients (age 18–25) than in pediatric patients (age < 18), and the older patients were more inclined to experience any-grade CRS or neurotoxicity, whereas the efficacy profile was also generally similar across age groups [6]. These results further confirmed the favorable efficacy of Tisa-cel with low toxicity in the real-world setting.

At the 2021 ASH annual meeting, several studies focused on the risk factors and predictors of relapse after CAR-T treatment, especially for high-risk patients. The risk of lineage switch (LS) following CD19-directed therapies in children and young adults with R/R B-ALL has been primarily limited to case reports. Of 420 CAR-T treated patients, after a median follow-up of 30.1 months, 2.9% (12/420) of patients experienced LS [7]. *KMT2A* rearrangement was presented in 75% (9/12) of patients with LS compared to 7.1% (20/408) of non-LS patients (*P* < 0.001). TP53 mutation was an independent prognostic factor of efficacy following CD19 CAR-T cell therapy. Among 64 B-ALL patients with TP53 mutation/chromosome 17p deletion who received CD19 CAR-T cell therapy, the presence of complex cytogenetics and not bridging into allogeneic hematopoietic stem cell transplantation (allo-HSCT) were two adverse factors affecting the long-term efficacy [8].

#### Novel strategies of CAR-T cell therapy to improve efficacy and overcome relapse

Although CD19-targeted CAR-T cell therapy has shown encouraging CR rate, up to 45% of patients eventually relapse, especially in adult B-ALL population [9]. Early (< 6 months from infusion) loss of B cell aplasia (BCA) is associated with high relapse risk [10]. In a single center experience, the maintenance therapy after CD19 CAR-T treatment achieved similar rate of overall survival (OS) and event-free survival (EFS) while being better-tolerated and lower-cost compared to HSCT in pediatric patients with B-ALL [11].

For CD19-positive relapse, the replacement of murine-derived binding domains by human-origin ones in the CAR construct or CAR-T cell reinfusion are common treatment approaches [12]. In an oral report from University of Pennsylvania at the meeting, reinfusion of the same CAR-T product was investigated [13]. Ten patients with CD19-postive relapse reinfused the same CD19 CAR-T cell product, 50% (5/10) of patients achieved CR, and 2 of the subsequently experienced another CD19-positive relapse.

To overcome CD19-negative relapse, CAR-T cells with different targets to B-cell surface markers could be applied. Dual-target CARs against CD19 and another antigen, such as CD22 or CD20, have been extensively studied in clinical trials [14–19]. At the 2021 ASH annual meeting, Seattle Children's Hospital reported the safety and feasibility of SCRI-CAR19 × 22v2, a dual transduced patient-derived product with lentiviral vectors encoding for either a CD19- or CD22-specific 4-1BB CAR [20]. Among 10 of 11 patients obtained CR with negative MRD, grade 1 CRS presented in 45% (5/11) of patients. ICANS occurred in 5 patients, with all grade 1 but one single self-limited grade 3. However, they also found that the peak engraftment of CAR-T cells in vivo lied most frequently between day + 7 and + 14, and it was predominated by CD22 CAR-T cells, with minimal contribution of the dual and CD19 CAR-T cells. Optimizing transduction methods may be required for a more balanced product to maintain effective dual targeting. Co-administration of CD19 CAR-T cells and CD22 CAR-T cells in adult patients with R/R B-cell malignancies also exhibited high efficacy and low toxicity [21]. Of the 11 patients who received co-administration with two humanized autologous CAR-T cells targeting CD19 and CD22, 100% (11/11) achieved CR with negative MRD in a month after infusion. Of the 13 treated patients, all experienced grade 1 or 2 CRS, and only 2 patients had ICANS. Moreover, CD19/CD123 dual-target CAR-T cells and tri-specific CD19xCD20xCD22 CAR-T cells from Legend Biotech are also presented promising preclinical data for patients with B-ALL who relapsed from prior CAR-T cell therapies [22, 23].

### Updates of B-cell non-Hodgkin lymphoma (B-NHL) therapy from the 2021 ASH annual meeting

#### Large B-cell lymphoma (LBCL)

CD19-targeted CAR-T cell therapy has demonstrated significant efficacy and reasonable safety in patients with R/R diffusive LBCL (DLBCL), high-grade B-cell lymphoma (HGBCL), DLBCL arising from transformed follicular lymphoma (tFL), and primary mediastinal B-cell lymphoma (PMBCL) [24–28]. Three commercial CD19-targeted CAR-T products, including Axicabtagene ciloleucel (Axi-cel), Tisa-cel and Lisocabtagene Maraleucel (Liso-cel), have been approved for adult patients with R/R DLBCL, HGBCL, and FL-derived DLBCL, who have received two or more lines of systemic therapy. Real-world experiences with commercial CAR-T cell products published recently are summarized in Table 1.

**Table 1:** Clinically approved CD19 CAR-T cell in B-cell lymphoma and B-ALL: Real-world Data

Clinical trials (reference)	CIBMTR Registry [29]	CIBMTR Registry [6]	Descar-T French National Registry [32]	Descar-T Registry and Lysa Group [41]	US Lymphoma CAR-T Consortium [31, 109]	US Lymphoma CAR-T Consortium [40]
Study type	Non-interventional prospective	Non-interventional prospective	Retrospective	Prospective	Retrospective	Retrospective
Indication	R/R aggressive B-NHL	R/R ALL	R/R aggressive B-cell lymphoma	R/R MCL	R/R LBCL	R/R MCL
Follow-up (months)	15.8 months	21.5	7.9	3.3	32.4	3.0
Patients (n)	405	400	550	57	275	93
Age (years)	66 (54.3% of patients aged ≥ 65 years)	13.9 (aged < 25 years)	62	67	60	67
Prior HSCT (auto/allo)	NA	28.8%	48%	NA	35.3%	27%
Medium number of previous lines of therapy	3	3	3	3	3	3
Bridging therapy permitted	NA	NA	87.8%	87.2%	54%	65%
Median turnaround time between leukapheresis and infusion (d)	27	27	50	56	NA	NA
Response rate (ORR/CR)	ORR 59.4% CR 39.5%	CR 87.3%	NA	ORR 88% CR 61.9%	ORR 82% CR 64%	ORR 86% CR 64%
OS	1-year 60.3%	1-year 79.5%	NA	NA	1-year 68.5% 2-year 56.4% 3-year 52.2%	6-month 82.1%
PFS	1-year 33.5%	1-year 54.3%	NA	6-month 57.9%	1-year 47.4% 2-year 41.6% 3-year 37.3%	3-month 80.6%
Risk factors for response	NA	Age ≥ 18y; heavy pretreatment; disease burden	High LDH level at time of infusion; time to failure < 1 month after infusion	NA	age > 60; high LDH level at time of conditioning	NA
Grade ≥ 3 neutropenia	NA	22.3%	NA	NA	NA	NA
Grade ≥ 3 thrombocytopenia	NA	20.8%	NA	NA	NA	NA
CRS, any grade	47.7%	58%	NA	78.7%	91%	88%
ICANS, any grade	17.0%	27.3%	NA	48.9%	69%	58%

*NA:* Not applicable

At the 2021 ASH annual meeting, registry data from the CIBMTR Registry was updated in 405 R/R B-NHL patients enrolled in previous cohort who received commercial CD19 CAR-T products after a longer medium follow-up (15.8 months) [29]. The objective response rate (ORR) was 59.4% (95% CI, 54.1–64.5), and 39.5% achieved CR. Patients experienced favorable safety outcomes with grade ≥ 3 CRS in only 4.9% (18/365). In the long-term survival analysis of ZUMA-1, after a long follow-up period (≥ 4 years) among 101 patients, the median OS was 25.8 months, and the 4-year OS rate was 44%. Median EFS was 5.7 months, with a 24-month EFS rate of 38% (95% CI, 28–47) [30]. Results from the US Lymphoma CAR-T Cell Consortium are similar to the ZUMA-1 trial, despite that the former included patients who did not meet ZUMA-1 eligibility criteria based on comorbidities [31]. However, CAR-T cell therapy in R/R LBCL still faces challenges. In DESCAR-T, a French nationwide registry, which enrolled all patients post commercialized CAR-T cell therapy, 43.3% (238/550) of treated patients relapsed within a median follow-up period of 7.9 months [32]. Therefore, to improve CAR-T cell efficacy and achieve prolonged duration of response (DOR), the application of lenalidomide or programmed cell death protein 1 (PD-1) inhibitors is a promising maintenance therapy that improved the OS in patients with R/R DLBCL [33, 34].

The prognosis for patients with early R/R LBCL after first-line therapy remains poor. ZUMA-7 (NCT03391466), an international, randomized, phase 3 trial, enrolled 359 patients with second-line R/R LBCL treated by Axi-cel versus standard of care (SOC) treatment (high-dose chemotherapy with autologous stem cell transplantation (ASCT)) [35]. Among randomized patients, the ORR and CR rate were significantly higher in Axi-cel group than SOC group (ORR: 83% vs. 50%, CR: 65% vs. 32%, *P* < 0.0001]. The toxicity of Axi-cel was manageable and consistent with third-line Axi-cel therapy. Patient-reported outcomes in ZUMA-7 also showed that Axi-cel treatment led to clinically meaningful improvement in quality of life compared with SOC [36]. In a pivotal, global, randomized, multicenter, phase 3 TRANSFORM study, Liso-cel as a second-line therapy also demonstrated significant improvement in the CR rate (66% vs. 39%, *P* < 0.0001) and progression-free survival (PFS) (median PFS 14.8 vs. 5.7 months, *P* = 0.0001) compared with SOC [37]. However, the randomized, global, phase 3 BELINDA study, comparing Tisa-cel and SOC in patients with second-line R/R LBCL, revealed no significant difference of EFS between two groups. After a median follow-up of 10 months, the median EFS was 3 months (Hazard ratio [HR] 1.07; 95% CI, 0.82–1.40; P = 0.69) in both groups, while the ORR was 46% vs. 43% [38]. The different results among these three clinical trials may be attributed to diverse study design elements; For instance, no bridging therapy was allowed in TRANSFORM and ZUMA-7, whereas BELINDA study enrolled more aggressive patients with permission of bridging therapy and potential delay of Tisa-cel application. Details from these clinical trials are summarized in Table 2. Therefore, larger phase 3 clinical studies with longer follow-up period are warranted to determine the role of CAR-T cell therapy as a second-line treatment of R/R LBCL.

**Table 2:** Reports of CD19 CAR‐T cell therapy as second-line or first-line therapy for large B-cell lymphoma at the 2021 ASH annual meeting

Clinical trials (reference)	ZUMA-7 (axi-cel) [35]	TRANSFORM (liso-cel) [37]	BELINDA (Tisa-cel) [38]	ZUMA-12 (axi-cel) [39]
Study type	Phase 3, randomized, global CAR-T VS SOC	Phase 3, randomized, global CAR-T VS SOC	Phase 3, randomized, global CAR-T VS SOC	Phase 2, multicenter, single-arm
Indication	Second-line	Second-line	Second-line	First-line
Patients (n)	359	184	322	40
Age (median, years)	59 (21–81)	59 (20–75)	50% patients ≥ 65 years	61 (23–86)
Inclusion criteria	Aged ≥ 18 years with LBCL, ECOG PS 0–1, failure of first-line chemotherapy	Aged ≤ 75 years, eligible for ASCT, and with R/R LBCL within 12 months after first-line chemotherapy; ECOG PS ≤ 1 and adequate organ function	R/R NHL within 12 months after first-line chemotherapy	High-risk LBCL, defined by histology (double‑ or triple-hit status) or an IPI score ≥ 3, plus a positive interim PET after 2 cycles of chemotherapy
Bridging therapy permitted	No	No	Yes	NA
Response rate (ORR/CR)	ORR: 83% vs 50% CR: 65% vs 32%	ORR: 89% vs 48% CR: 66% vs 39%	ORR: 46% vs 43% CR: 28% vs 28%	ORR: 89% CR: 78%
OS	Not reached vs 35.1 months	NA	NA	12-month estimate: 91%
PFS	8.3 months vs 2 months	14.8 months vs 5.7 months	3 months vs 3 months	Not reached 12-month estimate: 73%
Grade ≥ 3 neutropenia	NA	82%	NA	68%
Grade ≥ 3 thrombocytopenia	NA	58%	NA	NA
Grade ≥ 3 CRS	6%	1.1%	5%	8%
Grade ≥ 3 ICANS	21%	0	3%	23%
Follow-up (months)	24.9	6.2	10	15.9

*NA* not applicable

CD19 CAR-T cell is now being explored to serve as a first-line therapy for patients with high-risk LBCL. ZUMA-12, a phase 2, multicenter, single-arm study of Axi-cel, is trying to extend the indication of CD19 CAR-T cell therapy [39]. After a median follow-up of 15.9 months, 90% of (36/40) all patients had an objective response, and 80% (32/40) achieved CR. The estimated OS and PFS at 12 months were 91% and 75%, respectively. Eighty-five percent of patients (34/40) had grade ≥ 3 AEs, most commonly with cytopenias (68%, 27/40), ICANS (23%, 9/40), and CRS (8%, 3/40). These encouraging results demonstrate the feasibility of CAR-T cell therapy in the first-line treatment of high-risk LBCL.

#### Mantel cell lymphoma (MCL)

CAR-T cell therapy with Brexucabtagene Autoleucel (formerly KTE-X19) yields high response rates in R/R MCL and is now approved for patients with relapsed MCL in 2020 based on results from the pivotal ZUMA-2 study (NCT02601313). Real-world data from the US Lymphoma CAR-T Consortium demonstrated that the 6-month OS rate was 82.1% (95% CI, 57–75), and the 3-month PFS rate was 80.6% (95% CI, 54–71), while the incidences of CRS and ICANS were comparable to those reported in ZUMA-2 [40]. Other real-life studies from the Descar-T Registry and Lysa Group, and several US medical centers also supported the use of KTE-X19 in patients with R/R MCL who failed after BTK inhibitor treatment, including those high-risk patients (Table 1) [41, 42]. Longer follow-up is required to confirm the DOR.

#### Indolent non-Hodgkin lymphoma (iNHL)

Follicular lymphoma (FL) is an indolent disease with a continuous relapse pattern that typically requires multiple lines of therapy. At the meeting, primary analysis of the single-arm, multicenter, phase 2 ELARA trial enrolling 94 R/R FL patients demonstrated that, after a 17-month median follow-up, Tisa-cel produced high ORR (86.2%) and CR rate (69.1%) [43]. Durable response was also achieved in high-risk patients with progression of disease within 2 years (POD24) (CR 59%, 12-month PFS 60.8%). Safety was consistent with known Tisa-cel profile.

ZUMA-5 is a phase 2, multicenter, single-arm study that evaluated Axi-cel for treatment of R/R iNHL (including FL and marginal zone lymphoma (MZL)) [44]. Updated results showed that among 86 eligible patients with FL, the ORR was 94%, and the CR rate was 79% after a median follow-up of 30.9 months (range, 24.7–44.3) [45]. The estimated median DOR and PFS were 38.6 months and 39.6 months, respectively. Among 24 eligible patients with MZL, the median follow-up was 23.8 months (range, 7.4–39.4), with 83% ORR and 63% CR rate. Common grade ≥ 3 AEs in all treated patients with iNHL included neutropenia (33%), decreased neutrophil count (28%), and anemia (25%).

#### Novel strategies of CAR-T cell therapy to improve efficacy and overcome relapse

Although CD19-targeted CAR-T cell therapy has represented a paradigm shift in R/R B-NHL, many patients subsequently experienced disease progression or relapse with poor prognosis. Antigen escape or lack of adequate antigen expression contributes to the failure of CAR-T cell therapy [46]. CD22 and CD20, the other two lineage specific markers in B cell development, are attractive targets [19]. At the meeting, a single-center, phase 1 dose-escalation clinical trial (NCT04088890) revealed that CD22 CAR-T cell therapy mediated high durable remission rates in 21 patients with DLBCL who relapsed after prior CD19 CAR-T treatment. The ORR at day 28 was 86% (CR, n = 11; partial response (PR), n = 7), while the median PFS and OS were not reached after a mean follow-up of 7.3 months (range, 1.2–21.3) [47]. In another ongoing phase 1/2 clinical trial of CD20 CAR-T cell therapy for high-risk B-NHLs and chronic lymphocytic leukemia (CLL), the ORR was 94% (15/16) with CR rate of 62% (10/16), while no grade 3 or 4 CRS or ICANS were noted [48].

Targeting two antigens simultaneously may overcome antigen escape in B-cell malignancies. In a phase 1/2 single-center, prospective trial (NCT04186520), an IL7/IL15-expanded bispecific lentiviral anti-CD20, anti-CD19 (LV20.19) CAR-T cell product was tested [49]. Among 22 patients with DLBCL, FL, and MCL, the ORR was 91% (20/22), and the CR rate was 55% (12/22). Only one patient experienced grade 3 CRS, and 3 patients had grade 3 ICANS.

R/R NHL patients with TP53 gene disruption confer inferior prognosis. A clinical trial enrolled 60 patients with TP53 alteration who received CAR19/22 T-cell cocktail therapy [50]. After a median follow-up of 16.7 months (range, 3.1–41.0), the median OS was not reached, and the median PFS was 14.8 months (95% CI, 5.1–not estimated (NE)) in the patients harboring TP53 alterations. The estimated 24-month PFS and OS rates were 48.4% and 56.3%, respectively. Notably, the PFS and OS rates were similar between the patients with or without TP53 alterations when treated with CAR19/22 T-cell cocktail therapy, indicating that CAR19/22 T-cell cocktail therapy could overcome the negative impact of TP53 alterations in these patients. Among 28 patients with TP53 alterations and treated by ASCT incorporating CAR19/22 T-cell cocktail therapy, the estimated 24-month OS rate (89.3%, 95% CI, 70.4%–96.4%) and PFS (77.5%, 95% CI, 56.5%–89.3%) were higher than those of patients treated with CAR19/22 T-cell cocktail therapy alone. Therefore, ASCT combined with CAR-T cell therapy may be a potentially feasible strategy for treatment of high-risk lymphoma patients.

### Update of T-cell acute lymphoblastic leukemia (T-ALL) and T-cell lymphoma therapy from the 2021 ASH annual meeting

Compared with the significant progress of CAR-T cell therapy in B-ALL and B-NHL, broadening the success to treating T cell malignancies, including T-ALL and T cell lymphoma, is still intractable. Treating T-cell malignancies with T cell therapy presents a unique problem, since cytotoxic cell may share the same antigens with malignant cell, causing risks for the fratricide of cytotoxic cells and the contamination of malignant cell during manufacturing in the autologous setting [51, 52]. To avoid this problem, shared antigens such as CD7 can be knocked out of the CAR-T cell to specifically target CD7 on the malignant cell.

**Fig. 1:**
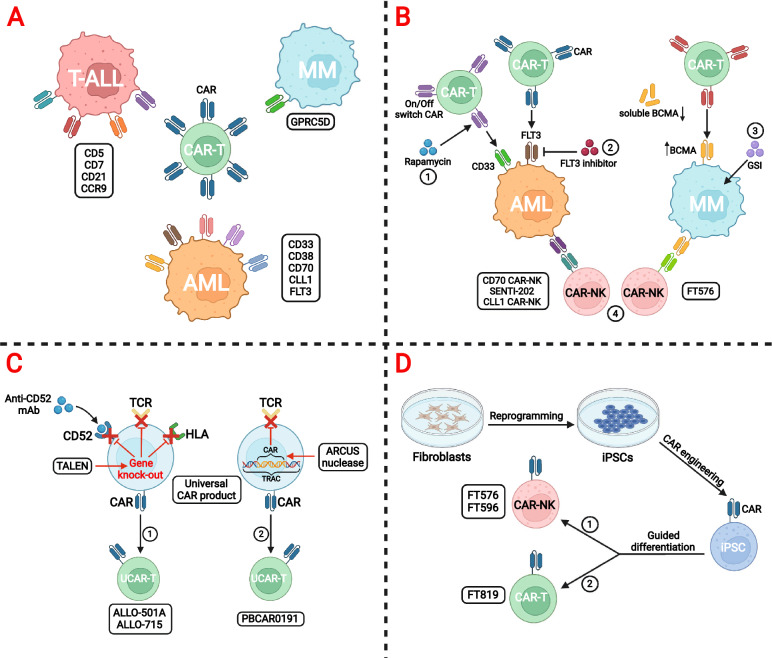
Progress of chimeric antigen receptor (CAR)-based cellular immunotherapy highlighted at the 2021 ASH annual meeting. **A** The exploration of novel antigen targets in T cell acute lymphoblastic leukemia (T-ALL), acute myeloid leukemia (AML), and multiple myeloma (MM). **B** Novel treatment strategies of CAR-based combinational therapy for AML and MM. (1) Pharmacologically controlled CD33-targeted anti-AML CAR-T product regulated by low concentrations of rapamycin. (2) Dual targeting with FLT3 CAR-T cell and FLT3 inhibitor for FLT3-mutant AML. (3) The combination of gamma secretase inhibitor (GSI) to modulate B cell maturation antigen (BCMA) expression improves efficacy of CAR-T cell therapy in MM. (4) Application of CAR-NK cell therapy in AML and MM. **C** The universal CAR-T cell therapy. (1) The combination of anti-CD52 mAb and TALEN-mediated gene-editing in universal CAR-T (UCAR-T) cell. (2) The disruption of T cell receptor (TCR) with CAR gene knock-in by ARCUS nuclease in UCAR-T cell. **D** Induced pluripotent stem cell (iPSC) as an innovative cell source for CAR-based cellular immunotherapy. (1) The production of iPSC-derived CAR-NK cell. (2) The production of iPSC-derived CAR-T cell

At the meeting, more than 10 reports from preclinical and clinical trials with novel targets like CD7, CD5, CD21 and CCR9 were published (Fig. 1A). Table 3 illustrates the outcomes of phase 1 clinical trials of CAR-T therapy for T-ALL and T cell lymphoma. In a phase 1 clinical trial of patient or donor-derived CD7-targeting CAR-T cell therapy for R/R T-ALL (NCT04572308), 17 patients were enrolled. After a median follow-up of 105 days (range, 32–206), 92.9% (13/14) of patients achieved CR, or CR with incomplete hematological recovery (CRi), and negative MRD [53]. In addition, 80% (4/5) of patients with extramedullary disease (EMD) also achieved extramedullary remission at a median of day 32 after infusion. Another phase 1 clinical trial of patient or donor-derived CD7-targeting CAR-T cell therapy for R/R T-cell lymphoblastic lymphoma (T-LBL) (NCT04916860) also showed a high initial efficacy and a good safety profile [54]. Five patients who had prior bone marrow blasts all achieved CRi with negative MRD. Of the 7 patients who had diffuse EMD, 5 achieved EMD CR. Preclinical data from an “off-the-shelf” allogeneic CD7-targeted CAR-T cell therapy WU-CART-007 also supported the well-tolerance and anti-tumor efficacy in vivo [55, 56]. Co-culture experiments in vitro confirmed strong cytotoxicity against CD7-expressing cells including T-ALL cell, primary T cell, and NK cell. Phase 1/2 studies are ongoing to further verify its efficacy and safety.

**Table 3:** Published clinical trials of CAR-T therapy for T-ALL and T-LBL at the 2021 ASH annual meeting

Clinical trials (reference)	ChiCTR2000034762 [110]	Abstract 473 [53]	Abstract 652 [54]	RD13-01 [111]	Abstract 654 [57]
Phase	Phase 1	Phase 1	Phase 1	Phase 1	Phase 1
Diseases	CD7-positive R/R T-ALL	R/R T-ALL	R/R T-LBL	R/R CD7^+^ hematological malignancies	R/R mature T-cell lymphoma
Target	CD7	CD7	CD7	CD7	CD5
Cell source	Stem-cell transplantation donors or new donors	Autologous	Autologous (7/8) or donor (1/8)	Universal CAR-T cells	Autologous
Patients (n)	20	17	8	11	9
Median of prior lines of therapies	3	5	5	NA	5
Prior HSCT	35%	17.6%	25%	25%	55.6%
Follow-up time	Median 6.3 months	Median 105 days	Median 93 days	Median 100 days	NA
Response rate (ORR/CR)	ORR: 95% CR: 90%	CR/CRi: 92.9%	CR/CRi: 62.5%	ORR: 82% CR (leukemia): 75% CR (T-NHL): 33.3%	ORR: 44.4% CR: 22.2%
CRS, any grade	100%	100%	100%	83.3%	44.4%
ICANS, any grade	15%	5.9%	12.5%	0	11.1%
Grade ≥ 3 neutropenia	100%	NA	NA	NA	NA
Grade ≥ 3 thrombocytopenia	100%	NA	NA	NA	NA
GvHD, any grade	60%	NA	NA	0	NA

*NA* not applicable

However, CD7-targeted CAR-T cell therapy for T-cell malignancies is challenging since target antigens are expressed on normal T cells. This leads to two main problems: loss of essential normal T cells and self-kill 'fratricide' of CAR-T cells. Therefore, exploring new targets in R/R T cell malignancies is inevitable. In a phase 1 clinical trial (NCT03081910) from the meeting, autologous T cells expressing a CD5-specific CAR were designed for patients with refractory or relapsed T cell lymphoma [57]. Forty-four percent (4/9) of patients responded, with 3 proceeding further allo-HSCT. After a long-term follow-up, 2 patients remained alive with CR for 29 months and 24 months, respectively. CCR9 is a G protein coupled receptor (GPCR) for the natural ligand CCL25, and is expressed in gut intraepithelial γδ T cells, some plasmacytoid dendritic cells, and double-positive thymocytes, but in less than 5% of normal circulating T and B cells. Potent anti-leukemic function of anti-CCR9 CAR-T cell has been proved both in vitro and in animal models, whose efficacy is not associated with loss of essential normal T cells or with CAR-T cell fratricide [58]. More clinical trials with long-term follow-up are needed to further evaluate the potential benefits and side effects of CAR-T cell therapy for T-cell malignancies.

### Update of multiple myeloma therapy from the 2021 ASH annual meeting

MM is an incurable hematological malignancy of plasma cells [59, 60]. Although many novel therapies are available for MM [61, 62], the treatment of MM still remains elusive. CAR- T cell therapy represents a promising immunotherapeutic approach with remarkable potential in relapsed and refractory multiple myeloma (RRMM) [63, 64]. Sequential CAR-T cell infusion followed by lenalidomide maintenance after ASCT sustained MRD negativity for more than 2 years in patients with newly diagnosed MM [65]. BCMA is currently the main target for CAR-T cells in MM, as it is predominantly expressed on differentiated malignant plasma cells [66]. In March 2021, FDA approved Idecabtagene Vicleucel (Ide-cel) as the first BCMA-directed CAR-T cellular immunotherapy for RRMM based on results from the pivotal KarMMa trial [67]. Recently, the FDA authorized another BCMA CAR-T product Ciltacabtagene Autoleucel (Cilta-cel) for patients with MM who relapsed at least after four prior lines of treatment.

#### Novel target of CAR-T cell therapy for RRMM

BCMA-targeted CAR-T cell therapy has shown promising results in RRMM, but relapse is common. Additional treatment options with novel therapeutic targets are warranted (Fig. 1A). In a phase 1 clinical trial of MCARH109, the first-in-class GPCR Class C Group 5 Member D (GPRC5D)-targeted CAR-T cell therapy, 12 patients with RRMM were treated [68]. The ORR was 83% (10/12), with 2 patients achieved stringent complete response (sCR). After a median follow-up of 13 weeks (range, 2.0–39.1), 75% (9/12) of patients achieved progression free without additional treatment. CRS occurred in 92% (11/12) of patients, with grade 3 in only one patient, and no ICANS events or dose limiting toxicities were reported. More importantly, 6 patients who relapsed after previous BCMA CAR-T cell therapy all responded to GPRC5D-targeted CAR-T cell therapy, including 2 patients who achieved sCR. Therefore, GPRC5D-targeted CAR-T cell therapy will be another potential treatment option for patients with RRMM, especially for those who relapsed after BCMA CAR-T cell therapy.

#### Updated clinical data for BCMA-targeted CAR-T cell therapy for RRMM

Long-term safety and efficacy data from diverse BCMA-targeted CAR-T cell products were updated at the 2021 ASH annual meeting, including Cilta-cel [69], CT053 [70], and others (Table 4). Cilta-cel is a CAR-T product with two BCMA-targeted single-domain antibodies from the Legend Biotech, China. At the meeting, clinical data from the phase 1b/2 CARTITUDE-1 study was updated [69]. After a long-term median follow-up of 18 months among 97 patients, the ORR was 97.9% (95% CI, 92.7–99.7); 94.8% (92/97) of patients achieved very good partial response (VGPR), or better, 80.4% (78/97) patients achieved sCR. The median DOR was 21.8 months (95% CI, 21.8–NE). The 18-month OS and PFS rates were 80.9% (71.4–87.6) and 66.0% (54.9–75.0), respectively. Of the 61 patients with CR evaluable for MRD, 91.8% (89/97) were MRD-negative at the 10^–5^ threshold; 44.3% (27/61) of patients sustained MRD 10^–5^ negativity ≥ 6 months, while only 18% (11/61) of patients maintained ≥ 12 months. Patients with RRMM who have triple class exposure to immunomodulatory drugs (IMiDs), proteasome inhibitors (PIs), and anti-CD38 monoclonal antibodies present a poor prognosis. Outcomes for patients with triple-class exposed RRMM in CARTITUDE-1 showed significantly improved ORR, CR rate, PFS, and OS compared to current real-world clinical practice [71]. CT053, a fully humanized BCMA CAR-T cell product, achieved deep and durable response in Chinese subjects with heavily pretreated RRMM, with a high MRD-negative sCR rate of 78.6% (11/14) and an acceptable safety profile [72]. Further investigations of Cilta-cel are ongoing in earlier lines of therapy and in outpatient settings (CARTITUDE-2 [NCT04133636], CARTITUDE-4 [NCT04181827], and CARTITUDE-5 [NCT04923893]) [69, 73, 74].

**Table 4:** Recent updates of CAR‐T cell therapy for multiple myeloma at the 2021 ASH annual meeting

Clinical trials (reference)	MCARH109 [68]	Ide‐Cel (KarMMA) [67]	Cilta‐Cel (CARTITUDE‐1) [69, 112]	Cilta‐Cel (CARTITUDE‐2) [73]	CT053 (Lummicar and CG study) [70]	CT103A [76, 77]	bb21217 (CRB‐402) [75]
Phase	1	2	1b/2	2 Cohort B	1	1/2	1
Patients (n)	12	128	97	18	38	71	72
Target	GPRC5D	BCMA	2‐epitope BCMA	2‐epitope BCMA	BCMA	BCMA	BCMA with PI3Ki bb007
Medium number of previous lines of therapy	8	6	6	1 (early relapse after initial therapy)	≥ 2	4	6
Prior ASCT	NA	94%	NA	77.8%	NA	28.2% (18.3% with previous CAR-T therapy)	NA
Response rate (ORR/CR)	ORR 83%	ORR 73% CR 33%	ORR 97.9% CR 80.4%	ORR 88.9% CR 27.8%	ORR 92.1% CR 78.9%	ORR 96% CR 54%	ORR 69% CR 28%
OS	NA	Median 19.4 months	18-month 80.9%	NA	NA	NA	NA
PFS	13-week 75%	Median 8.8 months	18-month 66%	NA	Median 22.7 months	NA	NA
CRS, any grade	92%	84%	94.8%	83.3%	73.7%	93%	75%
ICANS, any grade	0	18%	21%	5.6%	0	1.4%	15%
Grade ≥ 3 neutropenia	NA	89%	94.8%	NA	NA	NA	NA
Grade ≥ 3 thrombocytopenia	NA	52%	59.8%	NA	NA	NA	NA
Follow-up (months)	3.0	13.3	18	4.7	13.9	4.9	9

*NA* not applicable

#### Modified BCMA-target CAR-T therapy for RRMM

Many approaches for optimization of BCMA-targeted CAR-T cell therapy are under exploration and development. Bb21217 has the same CAR molecule as that in bb2121 (Ide-cel), but with a PI3K inhibitor motif bb007 added to the CAR construct during ex vivo culture to enrich the memory-like T cells in drug product and to decrease the proportion of highly differentiated or senescent T cells. Updated results from the phase 1 CRB-402 study showed that among 72 patients with RRMM, the ORR was 69% (50/72) and sCR was achieved in 28% (20/72) of patients [75]. Of the 15 patients evaluable for MRD with ≥ CR, 93% (14/15) of patients were MRD-negative. Eighty-one percent (30/37) of patients and 60% (9/15) of patients maintained detectable CAR-T cells at 6 and 12 months, respectively. The study also revealed that patients with higher levels of proliferative, less differentiated, memory-like CAR-T cells at peak expansion were more likely to experience prolonged DOR. CT103A, a fully human BCMA-specific CAR-T product, showed excellent safety and promising efficacy in heavily pretreated RRMM patients [76]. The unique CAR structure containing fully human single-chain variable fragment (scFv) may bypass the potential host anti-CAR immunogenicity and retain antitumor activity. In a multi-center, single-arm, phase 1/2 study of CT103A, after a median follow-up of 147 days (range, 31–1029), 96.0% (48/50) of patients achieved ORR, with VGPR or better of 82% (41/50) [77]. For 13 patients previously treated with BCMA CAR-T cell therapy, the ORR was 76.9%, with CR rate ≥ 38.5%. Surprisingly, CT103A was still detectable in 88.5% (23/26) of patients at 6 months and 87.5% (14/16) of patients at 12 months after infusion. Another fully humanized anti-BCMA CAR-T product, CT053, is ongoing in the phase 1 investigator-initiated clinical studies (NCT03380039, NCT03716856, NCT03302403) for RRMM in China. From the abstract published at the meeting, the ORR and CR rate were 92.1% and 78.9% after a median follow-up of 13.9 months [70]. The ORRs for patients with EMD, high-risk cytogenetics, and ISS stage III were 91.7% (95% CI, 0.62–1.00), 83.3% (95% CI, 0.59–0.96), and 81.8% (95% CI, 0.48–0.98), respectively. AEs were consistent with known toxicities of conventional CAR-T cell therapies.

CAR density may influence antitumoral efficacy of BCMA CAR-T cell, which correlates with clinical outcomes. The combination of gamma secretase inhibitor (GSI) showed increased BCMA surface density on tumor cells and decreased soluble BCMA levels in the peripheral blood in an immunodeficient mouse model, therefore enhancing the efficacy of BCMA CAR-T cells (Fig. 1B). In a phase 1 first-in-human trial of BCMA-targeted CAR-T cells in combination with a GSI (JSMD194) for 18 patients with RRMM, all patients completed the 5-day run-in with JSMD194 [78]. The ORR was 89% (16/18), with 8 patients achieving CR. BCMA binding capacity increased from a median of 610 to 9563 receptors per cell. Therefore, GSI co-administration can increase BCMA surface density on plasma cells, augment anti-tumor activity of CAR-T cell, and induce durable and rapid response.

### Update of acute myeloid leukemia (AML) therapy from the 2021 ASH annual meeting

R/R AML patients have a dismal prognosis. Numerous tumor antigens, such as CD33, CD123, and CLL-1, have been explored as potential target antigens for treatment of AML in the past few years (Fig. 1A) [79–81]. However, due to the lack of ideal specific antigen targets and the risk of fatal “off-tumor, on-target” side effects, CAR-T cell therapy remains challenging in AML [82]. More than 20 preclinical and clinical studies of CAR-T cell therapies with different targets for AML patients were presented at the meeting. The most remarkable results are listed in Table 5.

**Table 5:** Selected preclinical and clinical studies of CAR‐T and CAR-NK cell therapy for acute myeloid leukemia at the 2021 ASH annual meeting

Clinical trials (reference)	Abstract 733 [113]	Abstract 408 (CYAD-02) [114]	Abstract 825 [83]	Abstract 905 (SC-DARIC33) [86]	Abstract 1691 [91]	Abstract 2799 (SENTI-202) [92]	Abstract 1725 [93]
Study type	Preclinical	Clinical	Clinical	Preclinical	Preclinical	Preclinical	Preclinical
Target	Preferentially Expressed Antigen in Melanoma (PRAME)	MICA/Micb	CD33	CD33	CD70	FLT3 and/or CD33	CLL-1
Cell source	T cells	T cells	T cells	T cells	Human peripheral blood NK cells	Allogeneic NK cells	Healthy donor peripheral blood NK cells
Disease	AML	AML/MDS	AML/MDS	AML	CD70 positive hematological and solid malignancies	AML	AML
Innovation	Target intracellular antigens by TCR mimic (mTCR) antibodies	Co-expression of shRNA with the NKG2D CAR	PRGN-3006 UltraCAR-T: non-viral gene delivery to simultaneously express CD33 CAR, membrane bound IL-15 (mbIL15) and kill switch; < 48 h without ex vivo expansion	Pharmacologically controlled by low dose of rapamycin	Non-viral TcBuster™ Transposon System; knockout of CD70 by CRISPR/Cas9 editing;	OR and NOT logic gated CAR gene circuit	Tc Buster (TcB) transposon system carrying a second generation CLL-1 CAR (CD28/CD3ζ or 41BB/CD3ζ) and hyperactive TcB transposase mRNA
Setting	In-vitro and in-vivo (mice)	Phase 1 first-in-human CYCLE-1 trial (NCT04167696)	Phase 1/1b first-in-human clinical trial (NCT03927261)	in-vitro and in-vivo (mice)	In vitro	In vitro	In vitro
Results	Anti-tumor reactivity of PRAME mTCR CAR-T cells can be enhanced by IFN-γ	7/11 patients: stable disease; good safety and tolerability	ORR 50% at dose level 1–3 × 10^5^/kg; good safety and tolerability	A phase 1 trial clinical trial is ongoing	Enhanced persistence of CAR-NK cells; resistant to fratricide	OR gate: increase AML tumor clearance to prevent relapse; NOT gate: protect healthy HSCs from off-tumor toxicity	Enhanced in vivo persistence and improved metabolic health by knocking out CISH gene using CRISPR/Cas9 editing

PRGN-3006 UltraCAR-T is a novel CAR-T product manufactured from autologous T cells within 48 hours, while using non-viral gene delivery system to simultaneously express CD33 CAR, membrane-bound IL-15 (mbIL-15), and suicide switch. In a phase 1/1b dose-expansion study of PRGN-3006 in adult patients with R/R AML, 15 heavily pre-treated patients received PRGN-3006 at different dose levels up to 1 × 10^6^ cells/kg [83]. The ORR for 6 patients at dose level (1–3 × 10^5^ cells/kg) was 50%. Among them, 2 patients with post-HSCT relapse also responded. Clinical therapeutic efficacy and safety of CD38-targeted CAR-T cells in AML patients who relapsed after HSCT were also investigated (NCT04351022) and revealed [84]. Four weeks after infusion, 66.7% (4/6) of patients achieved CR or CRi. The median OS and leukemia-free survival (LFS) were 7.9 months and 6.4 months, respectively. Side effects were clinically manageable among all patients. These novel CAR-T products may expand treatment options for patients with R/R AML.

Additionally, preclinical exploration is also important for the advancement of precise regulation and enhanced function (Fig. 1B). Dual targeting with FLT3 CAR-T immunotherapy and FLT3 inhibitor (gilteritinib) are promising therapeutic strategies in FLT3-mutant AML and *KMT2A*-rearrangement ALL [85]. SC-DARIC33, a pharmacologically controlled CD33-targeted anti-AML CAR-T product regulated by low concentrations of rapamycin, have been established and evaluated in an upcoming phase 1 clinical trial [86].

CAR-NK cell therapy has some considerable advantages over CAR-T cells, including unique recognition mechanism, powerful cytotoxic effect, and “off-the-shelf” nature [87, 88]. It is particularly attractive in AML since the inherent graft-versus-leukemia (GVL) reaction of NK cells can be effectively augmented by a CAR directed to an AML expressed antigen [89, 90]. The sources of CAR-NK cells include human peripheral blood NK cells, umbilical cord blood NK cells, or even NK cell lines. CAR-NK products targeting CD70 [91], CD33 or FLT3 [92], and CLL-1 [93] with different engineering methods, such as CRISPR/Cas9 or logic-gated CAR gene circuits, exhibit potent anti-leukemia activity in vitro and are now under preclinical evaluation (Fig. 1B). However, challenges of low persistence, low transduction efficiency, and immunosuppressive milieu of tumor microenvironment still exist.

### Update of the universal CAR from the 2021 ASH annual meeting

The universal “Off-the-shelf” allogeneic CAR-T cells and other immune effector cells, such as NK or gamma delta (γδ) T cells, can be premanufactured from healthy donors and may offer alternatives to autologous strategies. The main design strategy of universal CAR-T (UCAR-T) cells is to genetically modified allogenic CAR-T cells without immunogenicity, which has specific anti-tumor activity without graft-versus-host disease (GvHD) or host rejection (Fig. 1C).

The traditional strategy of “Off-the-shelf” CAR-T cell therapy is to develop a universal CAR-T product by disruption of the T-cell receptor alpha constant gene (TRAC) and CD52 gene to avoid GvHD. ALLO-501A is a genetically modified anti-CD19 allogenic CAR-T product that uses TALEN® technology to disrupt TRAC and CD52. In the updated phase 1/2 ALPHA2 (NCT04416984) study, 13 patients with R/R LBCL were enrolled with an additional anti-CD52 monoclonal antibody for selective and transitory host lymphodepletion [94]. The ORR and CR rate were 66.7% (4/6), with 3 patients PR converting to CR after consolidation. No CRS, ICANS, GvHD, or no dose-limiting toxicities occurred.

Another strategy with CD19 CAR knocked into the TRAC locus by a TRAC-specific ARCUS nuclease is also employed, which disrupts the endogenous T-cell receptor. PBCAR0191 is an allogeneic “off-the-shelf” CAR-T product targeting CD19 with T cells derived from non-HLA-matched healthy donors. In a phase 1/2 clinical trial, 21 patients with R/R B-cell malignancies were treated, including 16 NHL patients and 5 B-ALL patients [95]. Six patients progressed after treatment with auto-CD19 CAR-T cell, and 8 patients progressed after auto or allogeneic HSCT. Among the 13 evaluable patients with NHL, 8 patients (62%) achieved CR/CRi, while 4 out of 5 (80%) B-ALL patients obtained CR. PBCAR0191 also demonstrated a manageable safety profile with mild CRS and no evidence of GvHD [95, 96].

### Update of pluripotent stem cell-derived cellular therapy from the 2021 ASH annual meeting

While allogeneic CAR-T or CAR-NK cell therapies are already demonstrating clinical promise, these strategies remain limited due to donor variability and batch to batch heterogeneity [97]. It is desirable to utilize a single renewable cell source to standardize the manufacture and quality of these novel immunotherapies. Development of CAR-engineered induced pluripotent stem cells (iPSCs) has the potential to achieve true consistency and unlimited scalability [98]. Moreover, a single iPSC clone with relatively the best gene-editing efficiency can be selected and isolated to serve as a source for gene-edited cell bank.

Fate Therapeutics has developed FT819, a first-of-kind, allogeneic, off-the-shelf CAR-T product derived from iPSC line, which is precisely engineered to insert a novel anti-CD19 CAR into the TRAC locus, to achieve more regulated CAR expression and abrogate risk of GvHD [99]. FT819 is currently being used in a multi-center phase 1 study (NCT04629729) for treatment of R/R B-cell lymphoma, CLL, and precursor B-ALL [100].

CAR-engineered iPSC-NK cells are also under development. FT596 is an off-the-shelf iPSC-derived CD19 CAR-NK product for R/R B-cell lymphoma. FT596 incorporates three genetically encoded functional components: a CD19-targeted CAR; a novel high-affinity, non-cleavable CD16 Fc receptor that enhances tumor targeting and antibody-dependent cell cytotoxicity in combination with a therapeutic monoclonal antibody; and an IL-15/IL-15 receptor fusion that promotes cytokine-autonomous persistence [101, 102]. Twenty patients were enrolled and exhibited none dose-limiting toxicity or severe adverse events. Of the 17 efficacy-evaluable patients, 9 achieved an objective response after the first FT596 treatment cycle. At a single-dose level of ≥ 90 million cells, 8 of 11 efficacy-evaluable patients achieved an objective response, including 7 patients with CR [103]. FT576, a similar iPSC-derived CAR-NK product targeting BCMA, also showed efficiency in preclinical studies (Fig. 1B) [102]. Table 6, Fig. 1C, and Fig. 1D highlight some preclinical and clinical trials of universal CAR-T, CAR-NK products, and iPSC-derived immunotherapy at the 2021 ASH annual meeting.

**Table 6:** Selected preclinical and clinical trials of universal CAR-T, CAR-NK products, and iPSC-derived immunotherapy at the 2021 ASH annual meeting

Clinical trials (reference)	Abstract 649 ALLO-501A [94]	Abstract 302 PBCAR0191 [95]	Abstract 651 ALLO-715 [115]	Abstract 823 FT596 [103]	Abstract 1766 FT819 [100]
Study type	Single-arm, open-label, Phase 1/2 clinical trial (ALPHA2 Study, NCT04416984)	Phase 1/2 clinical trial	Open-label, Phase 1 trial (UNIVERSAL, NCT04093596)	Multicenter, Phase 1 clinical trial (NCT04245722)	Preclinical ongoing Phase 1
Target	CD19	CD19	BCMA	CD19	CD19
Cell source	Allogenic T cells	Allogenic T cells	Allogenic T cells	iPSC-derived NK cells	iPSC-derived T cells
Disease	R/R large B-cell lymphoma	CD19^+^ R/R B-ALL or NHL	R/R multiple myeloma	R/R B-cell lymphoma	B-cell malignancies
Innovation	TALEN® gene editing to disrupt TRAC and CD52 gene	CD19 CAR is knocked-into TRAC locus after editing with a TRAC-specific ARCUS nuclease	TALEN® gene editing to disrupt TRAC and CD52 gene	High-affinity, non-cleavable CD16 Fc receptor and IL-15/IL-15 receptor fusion	1XX anti-CD19 CAR is inserted under the regulation of TRAC locus
Patient (n)	15	16 NHL 5 B-ALL	42	20	NA
Response rate (ORR/CR)	ORR: 50% CR: 50%	NHL: ORR 85%; CR/CRi 62% B-ALL: ORR:80%; CR/CRi 80%	ORR: 61.5% VGPR + : 38.5%	ORR of whole cohort: 52.9%; single-dose levels of ≥ 90 million cells: ORR: 72.7%; CR: 63.6%	NA
CRS, any grade	0	NA	52.4%	10%	NA
ICANS, any grade	0	4.8%	2.4%	0	NA
GvHD	0	0	NA	0	NA

*NA* not applicable

### Mechanism research of CAR-T therapy from the 2021 ASH annual meeting

Although CAR-T cell therapy has shown high clinical efficacy in hematological malignancies, there is unpredictable variability in the duration and depth of response. The mechanisms behind these divergent outcomes are not well understood yet. Heterogeneity of patients at the level of both tumor genomics and tumor microenvironment (TME) likely contributes to this important knowledge gap.

Single-cell multi-omics can provide a better understanding of the dynamic and evolution of CAR-T cells in human body. At the 2021 ASH annual meeting, Zachary Jackson et al. employed single cell RNA sequencing (scRNA-seq) and protein surface marker profiling in serial CD19 CAR-T cell samples from patients with NHL [104]. They revealed the evolution of CAR-T cells toward a non-proliferative, highly-differentiated, exhausted state in patients with poor response at the transcriptional and translational levels. David T. Melnekoff et al. also performed a longitudinal high resolution single cell genomic and proteomic analysis for patients with MM treated by BCMA CAR-T cell therapy [105]. They found significant up-regulation of anti-apoptotic genes at baseline and at relapse in poor responders, suggesting a novel mechanism of tumor-mediated escape. Molecular fate mapping of long-term persisting CAR-T cells from two leukemia patients with CR over a decade revealed functional persistence of CAR-T cells as a key predictor for durable remission [106].

CRS is the most common severe toxicity associated with CAR-T cell therapy. Caroline Diorio et al. performed comprehensive secretome profiling to measure more than 1400 serum analytes in serial samples collected from patients treated by CD19 CAR-T cells [107]. Two novel pre-infusion biomarkers, MILR1 and FLT3, were identified to predict the development of CRS. FLT3/FLT3 ligand may play a potential biological role in severe CRS.

Modulation of the gut microbiota by using antibiotics can enhance the efficacy of tumor-specific T cells. In a retrospective cohort of patients with B-ALL receiving oral vancomycin after CD19 CAR-T cell therapy, higher CAR-T cell expansion and serum inflammatory cytokines were observed [108]. In preclinical models, vancomycin-mediated modulation of the gut microbiota achieved better anti-tumor effect via cross-priming and enhanced CAR-T cell expansion in tumor samples.

## Conclusions and perspectives

With the development of cutting-edge technologies in life science, cellular immunotherapy has achieved major impact on the treatment of hematological malignancies during the past decade. To date, six commercial CAR-T cell products have been approved by FDA for the treatment of R/R B-cell malignancy and MM. At the 2021 ASH annual meeting, real-world data from different countries and regions fully proved the efficacy and safety of CAR-T cell therapy for patients with multi-line treatment failure. CAR-T cell therapy has even advanced into first-line or second-line therapy for some high-risk patients with invasive B-cell malignancies. CD7 CAR-T products for T cell malignancies revealed exciting clinical efficiency with manageable safety profile. Novel targets of CAR-T cell therapy, such as CD33 for AML, GPRC5D for MM, as well as dual-target CAR-T products can provide more potential choices for heavily pre-treated patients. Universal CAR products with diverse gene-editing strategies, including allogenic CAR-T and CAR-NK, hold promise in early clinical trials. iPSC-derived immunotherapy highlights a future direction of cellular immunotherapy: clone selection, powerful gene-editing, unlimited cell sources, and precise manipulation. In-vivo induced CAR-T cells by nanocarriers loaded with CAR genes or gene-editing tools can potentially overcome the current limitations. Basic research provides a comprehensive knowledge of the dynamics of CAR-T cells and the interactions in TME. However, the underlying mechanisms of anti-tumor activity and exhaustion of CAR-T cell, and tumor relapse after treatment are very complicated and remain obscure. The combination with other treatment strategies, such as small molecule inhibitors or HSCT, may improve clinical outcomes. Further studies are warranted to comprehensively understand the advantages, efficacy, long-term complications, and major diversity among CAR-engineered cells.

## Data Availability

The material supporting the conclusion of this review has been included within the article.

## Abbreviations

AE: Adverse event
ALL: Acute lymphoblastic leukemia
AML: Acute myeloid leukemia
Allo-HSCT: Allogenetic hematopoietic stem cell transplantation
ASCT: Autologous stem cell transplantation
Axi-cel: Axicabtagene Ciloleucel
B-ALL: B-cell ALL
BCA: B-cell aplasia
BCMA: B cell maturation antigen
BOR: Best overall response
B-NHL: B-cell non-Hodgkin lymphoma
CAR: Chimeric antigen receptor
CAR-T: CAR T cell
CI: Confidence interval
Cilta-cel: Ciltacabtagene Autoleucel
CIMBTR: Center for international blood and marrow transplant research
CLL: Chronic lymphocytic leukemia
CR: Complete remission
CRi: CR with incomplete hematological recovery
CRS: Cytokine release syndrome
DLBCL: Diffusive large B-cell lymphoma
DOR: Duration of response
EFS: Event-free survival
EMD: Extramedullary disease
FL: Follicular lymphoma
GSI: Gamma secretase inhibitor
GPCR: G protein coupled receptor
GvHD: Graft-versus-host disease
GVL: Graft-versus-leukemia
HGBCL: High-grade B-cell lymphoma
HR: Hazard ratio
ICANS: Immune effector cell-associated neurotoxicity syndrome
Ide-cel: Idecabtagene Vicleucel
IMiD: Immunomodulatory drug
iNHL: Indolent non-Hodgkin lymphoma
iPSC: Induced pluripotent stem cell
LBCL: Large B-cell lymphoma
LFS: Leukemia-free survival
Liso-cel: Lisocabtagene Maraleucel
LS: Lineage switch
mbIL-15: Membrane-bound interleukin-15
MCL: Mantel cell lymphoma
MM: Multiple myeloma
MRD: Minimal residual disease
MZL: Marginal zone lymphoma
NE: Not estimated
NK: Natural killer
ORR: Overall response rate
OS: Overall survival
PD-1: Programmed cell death protein-1
PFS: Progression-free survival
PI: Proteasome inhibitor
PMBCL: Primary mediastinal B-cell lymphoma
POD24: Progression of disease within 2 years
PR: Partial response
R/R: Relapsed and/or refractory
RRMM: Relapsed and refractory multiple myeloma
scFv: Single-chain variable fragment
sCR: Stringent complete response
scRNA-seq: Single cell RNA sequencing
SOC: Standard of care
T-ALL: T-cell ALL
TCR: T-cell receptor
tFL: Transformed follicular lymphoma
Tisa-cel: Tisagenlecleucel
T-LBL: T-cell lymphoblastic lymphoma
TME: Tumor microenvironment
TRAC: T-cell receptor alpha constant gene
UCAR-T: Universal CAR-T
VGPR: Very good partial response
